# The intrinsically kinase-inactive EPHB6 receptor predisposes cancer cells to DR5-induced apoptosis by promoting mitochondrial fragmentation

**DOI:** 10.18632/oncotarget.12838

**Published:** 2016-10-24

**Authors:** Amr M. El Zawily, Behzad M. Toosi, Tanya Freywald, Vijaya V. Indukuri, Franco J. Vizeacoumar, Scot C. Leary, Andrew Freywald

**Affiliations:** ^1^ Department of Pathology and Laboratory Medicine, College of Medicine, University of Saskatchewan, Royal University Hospital, Saskatoon, SK, S7N 0W8, Canada; ^2^ Faculty of Science, Damanhour University, Damanhour, 22516, Egypt; ^3^ Cancer Research, Saskatchewan Cancer Agency, Saskatoon, SK, S7N 5E5, Canada; ^4^ Department of Biochemistry, College of Medicine, University of Saskatchewan, Saskatoon, SK, S7N 5E5, Canada

**Keywords:** EPHB6, DRP1, mitochondrial dynamics, cancer, DR5

## Abstract

Death Receptor 5 (DR5) is a promising target for cancer therapy due to its ability to selectively induce apoptosis in cancer cells. However, the therapeutic usefulness of DR5 agonists is currently limited by the frequent resistance of malignant tumours to its activation. The identification of molecular mechanisms that determine outcomes of DR5 action is therefore crucial for improving the efficiency of DR5-activating reagents in cancer treatment. Here, we provide evidence that an intrinsically kinase-inactive member of the Eph group of receptor tyrosine kinases, EPHB6, induces marked fragmentation of the mitochondrial network in breast cancer cells of triple-negative origin, lacking expression of the estrogen, progesterone and HER2 receptors. Remarkably, this response renders cancer cells more susceptible to DR5-mediated apoptosis. EPHB6 action in mitochondrial fragmentation proved to depend on its ability to activate the ERK-DRP1 pathway, which increases the frequency of organelle fission. Moreover, DRP1 activity is also essential to the EPHB6-mediated pro-apoptotic response that we observe in the context of DR5 activation. These findings provide the first description of a member of the receptor tyrosine kinase family capable of producing a pro-apoptotic effect through the activation of ERK-DRP1 signaling and subsequent mitochondrial fragmentation. Our observations are of potential practical importance, as they imply that DR5-activating therapeutic approaches should be applied in a more personalized manner to primarily treat EPHB6-expressing tumours. Finally, our findings also suggest that the EPHB6 receptor itself may represent a promising target for cancer therapy, since EPHB6 and DR5 co-activation should support more efficient elimination of cancer cells.

## INTRODUCTION

Deregulation of apoptotic responses is a frequent phenomenon in most types of malignancies and cancer cells are often resistant to apoptosis induction [1–3]. Surprisingly, two death receptors, DR4 and especially DR5 are strongly expressed on tumour cells [4–8]. Moreover, activation of these receptors with their common ligand, TRAIL, or with agonistic antibodies triggers apoptotic responses predominantly in cancer cells, while sparing normal ones [9, 10]. Based on these findings, DR4 and DR5 are considered to be promising targets for cancer therapy, and clinical studies with DR4 and DR5 agonists have been initiated. Unfortunately, while all clinical trials to date have confirmed the safety of DR4 and DR5 activating reagents, neither DR4 nor DR5 activation produced a statistically significant positive effect on unselected cancer patients [11]. This is at least partially due to the action of molecular mechanisms that affect the sensitivity of cancer cells to apoptotic signaling initiated by these receptors. DR4 and DR5 initiate apoptotic cell death through both the mitochondrial-independent, extrinsic and the mitochondrial-dependent, intrinsic pathways [7, 11, 12]. In agreement, several mechanisms that have the potential to impinge upon these pathways have been shown to suppress the responsiveness of cancer cells to DR4 or DR5 signaling. These include reduced expression of CASPASE-8 [13, 14], overexpression of the apoptosis-inhibiting proteins, BCL2, CIAP2 and MCL-1 [15, 16], and overactivation of the AKT signaling pathway [17]. In addition, cancer cells also reduce their responsiveness to DR4 and DR5 activation by decreasing the expression of these molecules or by reducing their abundance at the cell surface [18, 19]. While a detailed understanding of the molecular machinery that controls DR4 and DR5 action in cancer cells is crucial for improving the efficiency of DR4- and/or DR5- activating agents in cancer treatment, and for selecting patients with tumours that are more amenable to this therapeutic approach, surprisingly little is known about the mechanisms that improve the sensitivity of cancer cells to these molecules.

Previously published observations indicate that a member of the Eph group of receptor tyrosine kinases, EPHB4, suppresses TRAIL-induced apoptosis in some types of solid tumours, including breast cancer cells [20–22], indirectly suggesting that its signaling partners may also actively regulate this response in a positive or negative manner. Signaling of Eph receptors mostly depends on their inherent kinase activity, which assures the ligand-induced tyrosine phosphorylation that is required for interactions with cytoplasmic molecules and the activation of downstream signaling pathways [23–25]. Nevertheless, the Eph group possesses two kinase-deficient members, EPHA10 [26] and EPHB6 [27, 28], that are thought to play an important role in modulating biological responses initiated by their kinase-active relatives [25]. Interestingly, our previous work showed that the EPHB6 receptor suppresses aggressiveness of breast cancer cells by interacting with EPHB4 and interfering with EPHB4 action [29], which implies that EPHB6 may also enhance the DR4- or DR5- mediated apoptotic response. In our present work, we mostly focus on EPHB6 function in triple-negative breast cancer (TNBC) cells, since triple-negative breast tumours that miss expression of the estrogen and progesterone receptors, and do not overexpress HER2 represent the most aggressive breast cancer subtype, and a targeted therapy for the effective treatment of this lethal malignancy remains elusive [30]. Our investigation reveals a novel mechanism, where EPHB6 stimulates ERK-DRP1 signaling in TNBC cells, which in turn promotes mitochondrial fragmentation and enhances the DR5-induced apoptotic response. As such, we believe that these findings are of potential clinical importance, because they imply that DR5-targeting therapeutic approaches should be applied in a personalized manner mostly to patients with TNBC tumours that express EPHB6 and also suggest that simultaneous administration of EPHB6 and DR5 agonists is likely to assure more effective killing of TNBC cells. Our findings are also of general importance, since they provide to our knowledge the first description of a member of the receptor tyrosine kinase family capable of initiating a pro-apoptotic effect on mitochondria through the ERK-DRP1 signaling cascade.

## RESULTS

### The EPHB6 receptor enhances DR5-initiated apoptosis in TNBC cells

Previous reports, including the work of our group, have shown that some receptors of the EphB type suppress apoptotic responses triggered by death receptors [20-22, 31]. Since earlier work shows that EPHB6 often executes its responses through interactions with other EphB receptors [29, 32, 33], we examined if it also interferes with DR5-induced apoptosis in cancer cells. We focused specifically on DR5 action because breast cancer cells have been found to preferentially respond to DR5 rather than DR4 activation [34, 35].

**Figure 1:**
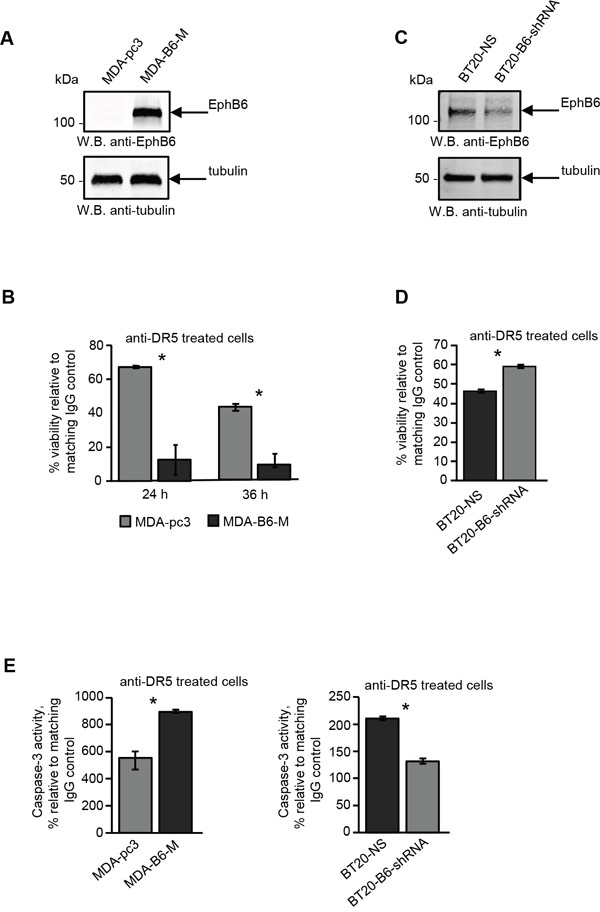
The EPHB6 receptor enhances DR5-induced cell death and CASPASE-3 activation in triple-negative breast cancer cells **A.** Western blot analysis of EPHB6 expression in triple-negative breast cancer (TNBC) cells, MDA-MB-231, stably transfected with the pcDNA3 expression vector encoding myc-tagged EPHB6 (MDA-B6-M) or mock-transfected with the empty vector (MDA-pc3). Western blotting with anti-tubulin was used as a loading control. **B.** Anti-DR5 activating antibody or matching non-specific IgG (both at 10 μg/mL) were immobilised overnight at 4°C on 96-well plates. MDA-B6-M and MDA-pc3 cells were loaded onto the pre-coated plates (5 × 10^4^ cells/well) in DMEM containing 0.5% FBS, and incubated at 37°C for 24 h or 36 h. To assess cell survival, cells were stained with resazurin and the fluorescence was measured using a SpectraMax M5 plate reader. Each graph represents the analysis of quadruplicates. The graphs present cell viability in DR5-treated cells as percentages relative to matching non-specific IgG controls. **C.** TNBC cells, BT-20, were transduced with EPHB6-targeting shRNA (BT20-B6-shRNA) or non-silencing shRNA (BT20-NS). EPHB6 expression was analysed by Western blotting as in (A). **D.** BT20-NS and BT20-B6-shRNA cells were stimulated in 96-well plates (1.5 × 10^4^ cells/well) for 36 h with anti-DR5 pre-immobilised at 20 μg/mL, as in (B). Matching non-specific IgG was used as a control. Cell survival was monitored by the MTT assay. A Spectramax 340PC plate reader was used to measure absorbance. Each graph represents the analysis of five replicates. The graph shows cell survival as a percentage relative to matching IgG controls. **E.** The indicated cells (1 × 10^6^ cells/well) were incubated in 6-well plates pre-coated with anti-DR5 or matching control IgG (both at 10 μg/mL) for 5 h and CASPASE-3 activity was measured using the EnzChek® CASPASE-3 Assay Kit #1 (Abcam) according to the manufacturer's instructions. Each graph represents the analysis of triplicates. The graphs present CASPASE-3 activity as percentages relative to IgG controls. All experiments were performed at least three times. *, P < 0.05, Student's t-test.

To assess the effect of EPHB6 on DR5-mediated apoptotic cell death, we partially silenced its expression in BT-20 TNBC cells and rescued it in TNBC cells, MDA-MB-231, which have lost endogenous EPHB6 expression due to promoter methylation [36]. Both EPHB6 silencing in BT-20 and rescue of EPHB6 expression in MDA-MB-231 cells were described in detail in our earlier publications [29, 37]. EPHB6-expressing and EPHB6-deficient cells from both TNBC lines were stimulated with an activating anti-DR5 antibody, and cell survival was monitored using resazurin staining or the MTT assay. This approach revealed that in contrast to the anti-apoptotic action of many other Eph receptors, EPHB6 significantly enhances the DR5-mediated death response in TNBC cells (Figure 1A-1D). Consistent with this observation, EPHB6 expression also potentiated DR5-induced activation of the effector CASPASE-3 (Figure 1E), further confirming the ability of EPHB6 to support DR5-triggered apoptotic action.

### The pro-apoptotic action of EPHB6 is associated with a fragmented mitochondrial network

**Figure 2:**
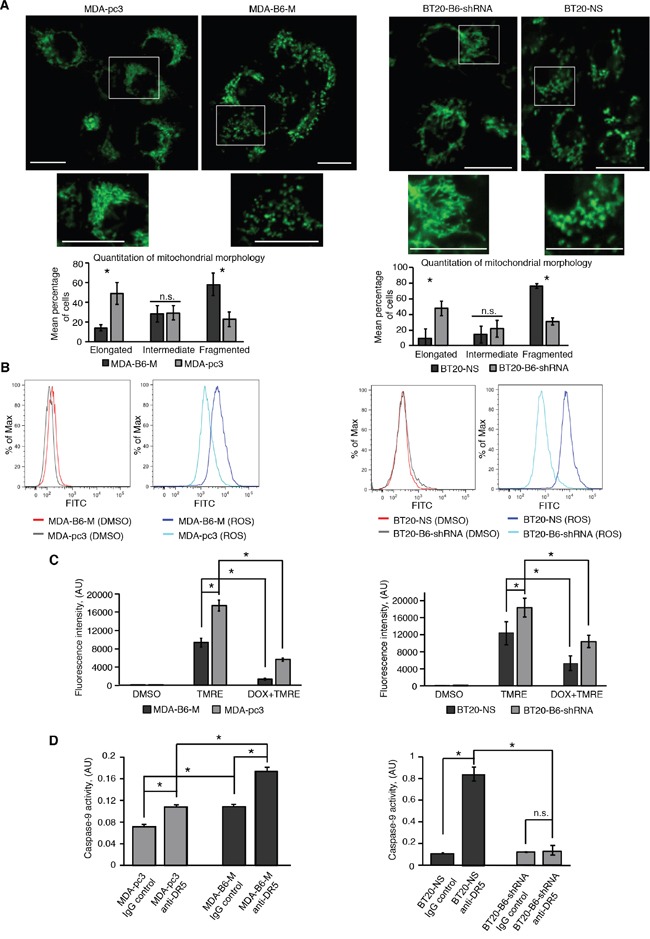
EPHB6 controls mitochondrial behaviour in TNBC cells **A.** To visualise mitochondria, the indicated cells were stained with 70 nM of MitoTracker Green for 20 minutes. Following staining, cells were washed twice with phenol red-free medium and live cell imaging was performed using a LSM 700 confocal microscope equipped with a live cell imaging chamber. Selected areas are shown at higher magnifications. Scale bar, 20 μm. Fluorescence intensity was adjusted using the Fiji software. The ZEN 2012 software was used to quantify mitochondrial morphology in at least 94 cells per experimental condition. Each graph represents the analysis of three independent experiments. **B.** The indicated cells were incubated with the ROS assay stain (total ROS assay 520 nm kit, affymetrIX eBioscience) or a matching volume of DMSO as a solvent control, and reactive oxygen species production was analysed by flow cytometry. **C.** To measure mitochondrial membrane potential, cells were incubated with 200 nM TMRE or a matching volume of DMSO for 25 min and fluorescence intensity was quantitated using a SpectraMax M5 plate reader. In addition, some cells were also pre-treated with 80 μM of doxorubicin (DOX) for 24 h to confirm the specificity of the TMRE staining, as DOX is expected to induce a strong intrinsic apoptotic response, causing a drop in mitochondrial membrane potential. Each graph represents the analysis of at least triplicates. **D.** Cells were treated with anti-DR5 or control IgG for 5 h, as in Figure 1E and CASPASE-9 activity was measured using the Colorimetric CASPASE-9 assay kit (Abcam) according to the manufacturer's instructions. A SpectraMax 340PC plate reader was used to quantitate CASPASE-9 activity. Each graph represents the analysis of triplicates. All experiments were performed at least three times. * *p<0.05*, Student's *t*-test. n.s., statistically not significant.

DR5 is known to use the mitochondrial-dependent pathway in its pro-apoptotic signaling [7, 11, 12]. Therefore, we used confocal microscopy to examine if the effects of EPHB6 on apoptosis are associated with altered mitochondrial dynamics, which reflects the balance between organelle fusion and fission events. Excitingly, our imaging experiments demonstrated that the mitochondrial network is disproportionately fragmented in breast cancer cells expressing the EPHB6 receptor even in the absence of DR5 activation (Figure 2A). Complementing its effect on mitochondrial fragmentation, EPHB6 also increased reactive oxygen species (ROS) production and caused a significant decrease in mitochondrial membrane potential (Figure 2B and 2C). The observed mitochondrial fragmentation is likely to be directly relevant to the pro-apoptotic action of EPHB6 because it is a response that precedes the final stages of apoptosis induction [38]. It is also known that mitochondrial fission is required for the initiation of molecular events that eventually trigger CASPASE-9 activation and ultimately lead to the activation of effector caspases, including CASPASE-3 [38]. Consistent with this model, EPHB6 enhanced DR5-mediated activation of both CASPASE-9 and CASPASE-3 (Figure 2D and Figure 1E), thus confirming the involvement of mitochondria in the pro-apoptotic function of the EphB6 receptor.

### The EPHB6 receptor relies on the activation of DRP1 for its pro-apoptotic effect on cancer cells

**Figure 3:**
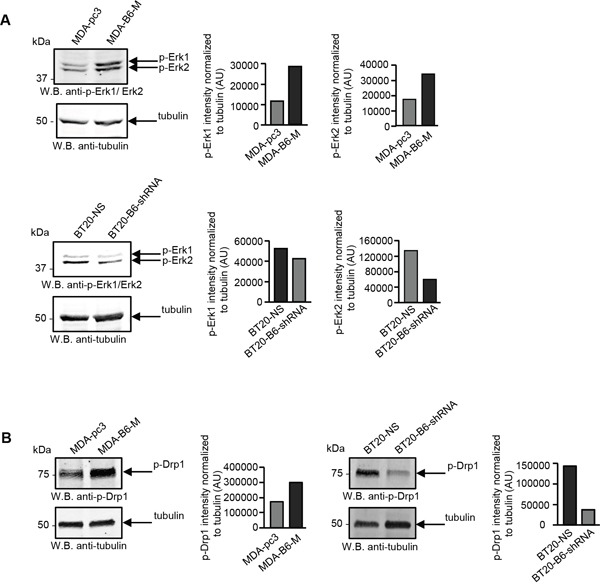
EPHB6 activates ERK kinases and DRP1 in cancer cells **A.** The indicated cells were lysed, cell lysates were resolved by SDS PAGE and phosphorylation of ERK1 and ERK2 kinases was analysed by Western blotting with anti-phospho-ERK (anti-p-ERK1/ERK2). ERK phosphorylation was quantitated by densitometry, measurements were normalized to tubulin controls and are presented in arbitrary units (AU). **B.** DRP1 phosphorylation on the activating residue, Ser616, was analysed by Western blotting with anti-phospho-DRP1 (anti-p-DRP1) as in (A). All experiments were performed at least three times.

**Figure 4:**
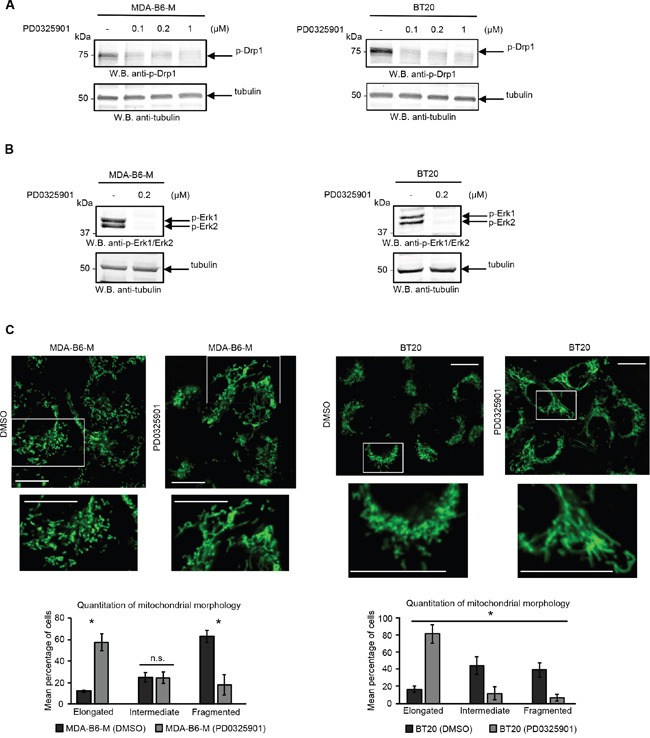
ERK activity is required for the EPHB6 effect on mitochondrial fragmentation **A.** Cells were treated for 24 h with a MEK inhibitor, PD0325901, at the indicated concentrations or with a DMSO volume matching the highest PD0325901 concentration (−) and DRP1 phosphorylation was assessed by Western blotting. **B.** To confirm the efficiency of MEK inhibition, cells were treated with 0.2 μM PD0325901 or a matching volume of DMSO (−) for 2 h and the phosphorylation status of ERK kinases was examined by Western blotting. **C.** Cells pre-treated for 24 h with 0.2 μM PD0325901 or DMSO were loaded with 70 nM MitoTracker Green for 20 min. Live cell imaging was performed using a LSM 700 confocal microscope. Selected areas are shown at higher magnifications. Scale bar, 20 μm. Quantification and analysis of mitochondrial morphology was done as in Figure 2A. All experiments were performed at least three times. * *p<0.05*, Student's *t*-test. n.s., statistically not significant.

**Figure 5:**
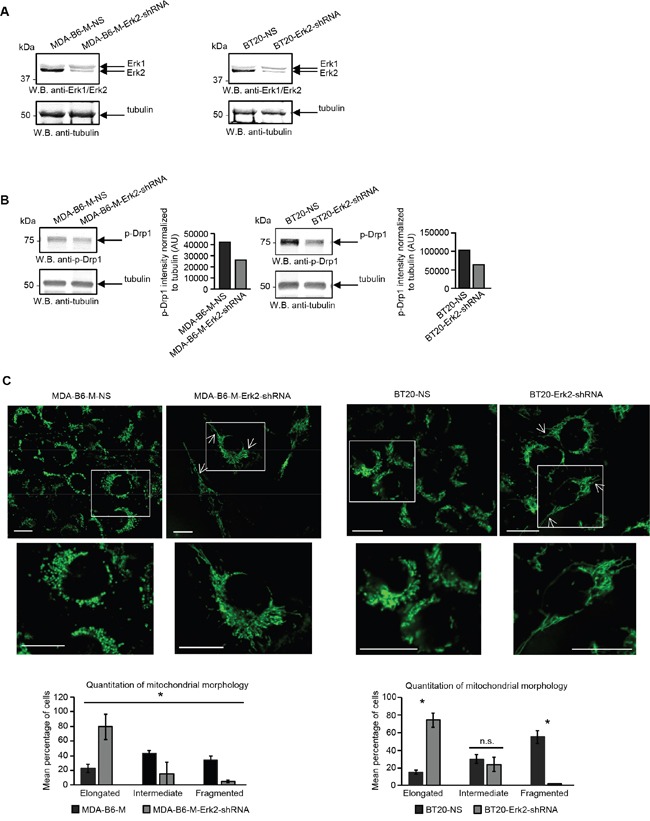
EPHB6 action is mediated by the ERK2 kinase **A.** MDA-B6-M and BT-20 cells were transiently transduced with ERK2-targeting shRNA (MDA-B6-M-ERK2-shRNA and BT20-ERK2-shRNA) or non-silencing shRNA (MDA-B6-M-NS and BT20-NS) and ERK2 expression was examined by Western blotting. **B.** Phosphorylation of DRP1 was analysed in the indicated cells by Western blotting. DRP1 phosphorylation was quantitated by densitometry, measurements were normalized to matching tubulin controls and are presented in arbitrary units (AU). **C.** Mitochondria were visualised in the indicated cells by confocal microscopy as in Figure 2A. Arrows indicate elongated mitochondria. Selected areas are shown at higher magnifications. Z-stack frames were split and fluorescence intensity was adjusted using the Fiji software. Scale bar, 20 μm. Quantitative analysis was done as in Figure 2A. Each graph represents two independent experiments. * *p<0.05*, Student's *t*-test. n.s., statistically not significant. All experiments were repeated at least twice.

**Figure 6:**
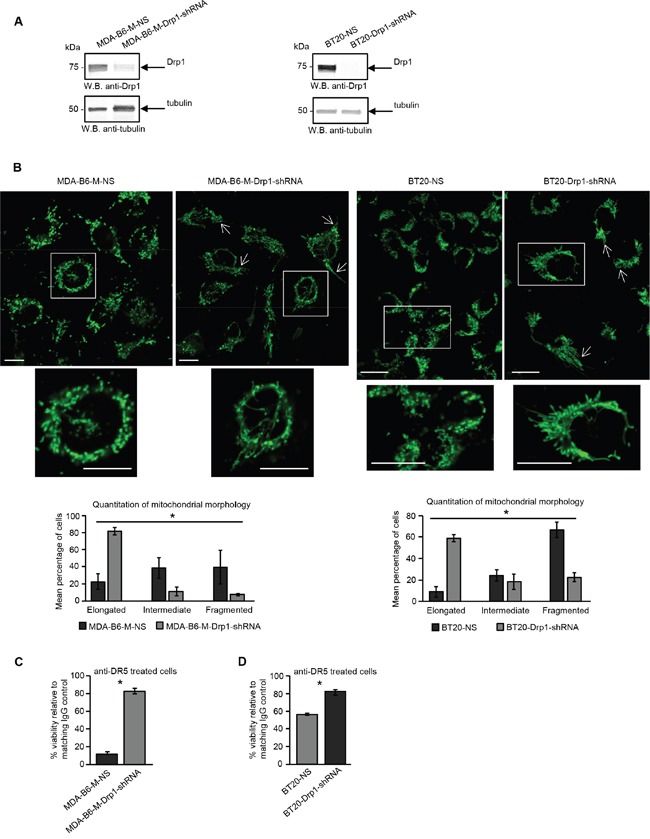
EPHB6 relies on DRP1 for its pro-apoptotic action **A.** MDA-B6-M and BT-20 cells were stably transduced with DRP1-targeting shRNA (MDA-B6-M-DRP1-shRNA and BT20-DRP1-shRNA) or non-silencing shRNA (MDA-B6-M-NS and BT20-NS) and DRP1 expression was examined by Western blotting. **B.** Mitochondria were visualised in the indicated cells by confocal microscopy as in Figure 2A. Arrows indicate elongated mitochondria. Selected areas are shown at higher magnifications. Z-stack frames were split and fluorescence intensity was adjusted using the Fiji software. Scale bar, 20 μm. Mitochondrial morphology was quantified as in Figure 2A. Each graph represents three independent experiments. **C.** Anti-DR5 or control IgG (10 μg/mL each) were immobilised overnight at 4°C onto 96 well plates. MDA-B6-M-DRP1-shRNA and MDA-B6-M-NS cells were loaded onto the pre-coated plates (3 x10^4^ cells/well), incubated for 24 h, and cell survival was monitored by resazurin staining. **D.** Anti-DR5 and control IgG were immobilised overnight onto 96-well plates at 20 μg/mL each. Cells (15,000/well) were seeded onto the pre-coated plates and incubated for 36 h. Cell survival was assessed by the MTT assay. The graphs in (C) and (D) represent cell viability as percentages relative to matching IgG controls. Each graph represents the analysis of quadruplicates. All experiments were performed at least three times. * *p<0.05*, Student's *t*-test.

There is considerable evidence that the fragmented mitochondrial network observed in many cancer cell types results from activation of the DRP1 GTPase [39, 40], which plays a central role in mitochondrial fission [41]. ERK2 is responsible for DRP1 activation through phosphorylation of Serine 616 [40] and it has recently been shown that the ERK1 and ERK2 kinases are both activated in MDA-MB-231 cells overexpressing EPHB6 [42]. Our recent observations also emphasize that ERK activation is suppressed in several breast cancer cell lines upon silencing of EPHB6 expression (Toosi et al, EMBO Molecular Medicine, in revision). We therefore examined if EPHB6 uses ERK-DRP1 signaling in its pro-apoptotic action. We confirmed the recently reported effect of EPHB6 on ERK kinases and found that in addition to ERK activation, EPHB6 expression also increases the amount of DRP1 that is phosphorylated on the activating residue, Serine 616. In contrast, silencing of EPHB6 expression produced the opposite effect in TNBC cells (Figure 3A and 3B). Inhibition of ERK signaling with a MEK inhibitor, PD0325901, blocked EPHB6-initiated DRP1 activation (Figure 4A and 4B) and suppressed mitochondrial fragmentation (Figure 4C) in two different TNBC cell lines, confirming an important role for ERK kinases in EPHB6 action. Consistent with these observations, silencing ERK2 expression by transducing EPHB6-expressing cells with an ERK2-specific shRNA (Figure 5A) resulted in a significant reduction in the abundance of phosphorylated DRP1 (Figure 5B) and a more reticular mitochondrial network (Figure 5C). Silencing of DRP1 expression with a DRP1-specific shRNA also blocked EPHB6-initiated mitochondrial fission, further demonstrating that the ERK2-DRP1 signaling pathway is essential for the effects of EPHB6 on mitochondrial dynamics (Figure 6A and 6B).

As mitochondrial fission represents a critical step in apoptosis induction [38] and the mitochondrial network is highly fragmented in EPHB6 expressing cells, we next directly addressed the role of DRP1 in the pro-apoptotic action of the EPHB6 receptor. Consistent with our initial observations that DRP1 is required for EPHB6-induced alterations in mitochondrial dynamics, silencing its expression efficiently suppressed the ability of EPHB6 to enhance DR5-mediated cell death (Figure 6C and 6D). Taken together, these findings provide strong support for an entirely novel model, where EPHB6 augments the pro-apoptotic effect of DR5 on TNBC cells by activating ERK2-DRP1 signaling, which in turn induces fragmentation of the mitochondrial network. Our model also provides a rational explanation for why in multiple cancer types, malignant tumours tend to reduce EPHB6 expression [37], since a decrease in EPHB6 levels should improve survival of cancer cells by protecting them from immunoelimination mediated by DR5 activation [43–45].

## DISCUSSION

It is now clear that the ability of malignant cells to regulate mitochondrial dynamics is critical to their oncogenic potential, yet our understanding of how mitochondrial shape and organelle function contribute to cancer-related phenotypes is only in its infancy [46]. Here, we show that EPHB6, an unusual member of the Eph group of receptor tyrosine kinases that lacks catalytic activity due to multiple alterations in the sequence of its kinase domain [27, 28], exerts a striking effect on the organization of the mitochondrial network in TNBC cells. EPHB6 expression promotes mitochondrial fragmentation by activating the ERK kinases, which leads to phosphorylation of an activating residue, Serine 616, in DRP1, a GTPase that plays an essential role in mitochondrial fission. According to previously published observations, the phosphorylated form of DRP1 assures membrane scission by assembling into an oligomeric, ring-like structure that physically constricts mitochondrial membranes [47, 48]. Our shRNA silencing experiments reveal that DRP1 phosphorylation in TNBC cells expressing EPHB6 is mediated at least in part by ERK2, a result that is consistent with a recent study demonstrating that the amino acids flanking Serine 616 comprise an evolutionarily conserved consensus sequence for ERK2 substrate recognition [40]. DRP1 function is tightly regulated by multiple post-translational modifications [49], and a recent study elegantly demonstrated that SUMOylation of DRP1 promotes cytochrome *c* release and apoptotic cell death via the intrinsic pathway [50]. The EPHB6 receptor also uses DRP1 activation to sensitize TNBC cells to pro-apoptotic stimuli mediated via the intrinsic pathway. EPHB6 expressing cells not only harbor a more fragmented mitochondrial network, but also produce more ROS and have a lower mitochondrial membrane potential. While these functional differences are not associated with an appreciable release of cytochrome *c* into the cytosol in unstimulated cells (data not shown), DRP1-induced fragmentation of the mitochondrial network in EPHB6 expressing cells does appear to make the organelle more vulnerable to pro-apoptotic signaling. Consistent with this idea, EPHB6 expression promotes the ability of a DR5 agonist to activate CASPASE-9, a signaling event that directly depends on the involvement of mitochondria in the apoptotic response. This effect ultimately enhances activation of the effector caspase, CASPASE-3, and causes a more efficient induction of cell death. Silencing of DRP1 effectively suppresses the apoptotic response to DR5 stimulation in EPHB6 expressing cells, suggesting a central role for this GTPase molecule in EPHB6 action. Interestingly, we have not observed the pro-apoptotic effect of EPHB6 expression in our experiments with paediatric T-cell acute lymphoblastic leukaemia (T-ALL) cells (data not shown), which further confirms the specificity of our observations in TNBC cells and indicates that the pro-apoptotic EPHB6 action via DRP1 activation may be restricted to certain types of malignancies. This distinction could be due to the fact that EphB receptors, including EphB6, act in T-ALL cells in a completely different molecular context, which collectively allows them to activate the AKT kinase, initiating anti-apoptotic signaling and supporting cell survival [31].

The results of our past studies [37] and those presented herein emphasize that whether TNBC tumours express EPHB6 should be a serious consideration with respect to choosing the most efficient therapeutic treatment options. Our previous work shows that EPHB6 is synthetic lethal with Src, and TNBC cells and tumours with EPHB6 deficiency are effectively eliminated by Src-inhibiting compounds [37]. Our current findings predict that these same tumour cells will be resistant to DR5 activation because they have relatively low levels of phosphorylated DRP1 and maintain a robust, reticular mitochondrial network. In contrast, TNBC tumours expressing EPHB6 are likely to have higher levels of the active, phosphorylated form of DRP1, a fragmented mitochondrial network and therefore be more sensitive to the DR5-initiated apoptotic signal. While a large body of evidence supports the idea that DRP1-mediated mitochondrial fission is pro-tumorigenic in nature [51–54], including in breast cancer [55], our findings clearly indicate that EPHB6-positive tumour cells should be more susceptible to DR5-activating therapeutic approaches. Importantly, this implies that EPHB6 may be used as a new biomarker for selecting TNBC tumours sensitive to DR5 activation and that DR5 agonists could produce better results if used selectively to treat EPHB6-positive tumours. In addition, our observations also highlight the potential for EPHB6 to be used as a novel target for cancer therapy. Thus, interventions that support its activity, including the application of stabilizing anti-EphB6 antibodies, with the simultaneous administration of DR5 agonists may improve tumour eradication. Future studies in animal models and in TNBC patients will be required to further validate the potential therapeutic application of these treatment approaches.

## MATERIALS AND METHODS

### Antibodies and reagents

Phospho-DRP1 (Ser616) and phospho-ERK antibodies were obtained from Cell Signaling Technology (Danvers, MA, USA). DRP1, β-tubulin and ERK1/2 antibodies were purchased from Santa Cruz Biotechnology (Dallas, TX, USA). Anti-EPHB6 was obtained from Santa Cruz and Sigma-Aldrich (St. Louis, MO, USA). Bovine serum albumin (BSA) was acquired from BioShop Canada Inc. (Burlington, ON, Canada). Dimethyl sulfoxide (DMSO), puromycin, doxorubicin and polybrene were purchased from Sigma-Aldrich. Resazurin was obtained from R&D Systems (Minneapolis, MN, USA). PD0325901 was acquired from Tocris (Bio-Techne, Minneapolis, MN, USA). Tween-20 was obtained from Fisher Scientific (Ottawa, ON, Canada).

### Cell culture

MDA-MB-231 and BT-20 TNBC cells were purchased from American Type Culture Collection (Manassas, VA, USA). MDA-MB-231 and BT-20 cells were grown in cell culture medium: DMEM supplemented with 1% penicillin/streptomycin, 1 mM sodium pyruvate and 10% FBS (all from Gibco, Life Technologies, Burlington, ON, Canada).

### Western blot analysis

Cell lysates were prepared by washing cells once with ice-cold phosphate buffered saline (PBS) and lysing them in lysis buffer (0.2% NP-40, 0.1 M EDTA, 0.3 M Tris, 0.1 M NaCl, 6 mM PMSF, and 3 mM sodium ortho-vanadate) for 30 minutes on ice. Cell debris was removed by centrifugation. Samples were resolved by SDS-PAGE, and transferred to a nitrocellulose membrane (Amersham, GE Healthcare Life Sciences, Baie d'Urfe, QC, Canada). Membranes were blocked in TBS (50 mM Tris Base, pH= 7.4, 150 mM NaCl) containing 0.1% Tween-20 and 5% BSA, or in PBS with 5% non-fat dry milk and 0.1% Tween-20, and then incubated with a primary antibody overnight at 4°C. Membranes were washed 3 times for 5 min with TBS or PBS, and incubated for 1 h with a fluorescently labeled secondary antibody in TBS or PBS containing 0.1% Tween-20 and 5% BSA or 5% non-fat dry milk. Following three additional washes with TBS or PBS, membranes were imaged with the LI-COR Odyssey imaging system (LI-COR Biotechnology, Guelph, ON, Canada). Fluorescence intensity was analysed by densitometry using the Carestream software (Carestream Molecular Imaging Software, New Haven, CT, USA). Figures were generated using the Carestream and PowerPoint software. Western Blot images were cropped using PowerPoint. Brightness and contrast of Western blot images were adjusted using the Carestream and PowerPoint software to optimize image presentation.

### Cell survival and caspase activation assays

Activating human Trail R2/TNFRSF10B (DR5) antibody (Cat # MAB631) from R&D Systems was used to induce apoptosis in TNBC cells. Anti-DR5 and matching non-specific control IgG (R&D Systems) were immobilized overnight at 4°C onto 96-well cell culture plates at 10 μg/mL or 20 μg/mL in DMEM supplemented with 0.5% FBS. Cells were then seeded in DMEM medium containing 0.5% FBS and incubated at 37°C for 24 or 36 h. The resazurin assay was used according to the manufacturer's instructions to assess cell survival and the fluorescence intensity was measured using a SpectraMax M5 plate reader (Molecular Devices, Sunnyvale, CA, USA). Alternatively, cell survival was monitored using the MTT (Thiazolyl Blue Tetrazolium Bromide) assay (Sigma- Aldrich). The MTT assay was performed according to manufacturer's instructions using a Spectramax 340PC plate reader (Molecular Devices).

To analyse CASPASE-3 or CASPASE-9 activation, 6-well plates were pre-coated with activating anti-DR5 or a matching non-specific IgG as described above. Cells were seeded onto the pre-coated 6-well plates (1 × 10^6^ cells/well) in DMEM medium containing 0.5% FBS and incubated at 37°C for 5 h. Caspase activity was monitored using the EnzChek® CASPASE-3 Assay Kit #1 (Cat # E-13183) or the CASPASE-9 Assay Kit, Cat# ab65608 (Abcam, Toronto, ON, Canada) by following manufacturer's instructions.

### Lentiviral transduction

Lentiviral particles encoding EPHB6-targeting shRNA or control non-silencing shRNA were purchased from Santa Cruz. Lentiviral particles encoding DRP1 shRNA (Sigma- Aldrich, Clone ID, NM_012062.3-1244s21c1) or ERK2 shRNA (Santa Cruz, Cat # sc-35335-SH) were generated by co-transfecting HEK 293T cells at ~80% confluence in 10 cm plates with the pMD2G (1.11 μg), pRSV-Rev (1.7 μg) and pMDLg/pRRE (1.7 μg) plasmids and 4.17 μg of either lentiviral vector in 10 mL of antibiotic-free DMEM containing 2% FBS and 30 μl METAFECTENE PRO (Biontex Laboratories, München, Germany). After 16 h, the transfection medium was replaced with regular culture medium. Viral particles were collected 48 and 72 h after transduction by filtration of the medium with 0.44 μm filters. Lentiviral particles were used to transduce cells by overnight incubation in medium containing 10 μg/mL polybrene (Sigma-Aldrich). The lentiviral transduction medium was replaced with cell culture medium and cells were incubated for additional 24-48 h. Cell lines with stable shRNA expression were generated by selection with 10 μg/mL of puromycin (Sigma-Aldrich) for 5 days. Western blotting was performed to confirm silencing of each target molecule.

### Reactive oxygen species (ROS)

ROS production was analysed by flow cytometry. Briefly, cells (10^6^ cells/sample) were incubated with DMSO or ROS assay stain solution for 1 h using the Total ROS assay kit 520 nm (affymetrIX eBioscience, San Diego, USA) following the manufacturer's instructions. Cells were harvested, centrifuged for 5 min at 300 relative centrifugal force, resuspended in PBS with ROS assay stain solution and incubated for 1 h at 37°C. Cell staining was assessed by flow cytometry. Results were analysed using the FlowJo software (FlowJo LLC, Ashland, OR, USA).

### Mitochondrial membrane potential

The tetramethylrhodamine ethyl ester (TMRE) kit (Abcam) was used to measure mitochondrial membrane potential. Cells were stained in 96-well plates (10^5^ cells/well) with 200 nM TMRE solution or a matching volume of DMSO as a control for 25 min at 37°C in an atmosphere of 5% CO_2_, following the manufacturer's instructions. MDA-pc3 and MDA-B6-M cells were stained as a monolayer, while BT20-NS and BT20-B6-shRNA were stained in suspension to optimize the staining. Cells were washed twice with PBS, resuspended in PBS with 0.2% BSA and TMRE fluorescence was measured using a SpectraMax M5 plate reader at Ex/Em = 549/575 nm.

### Immunofluorescence

Cells were plated on glass-bottom coverslip dishes (MatTek, AshLand, MA, USA) the day prior to imaging experiments. Cells were washed once with PBS and then incubated with 70 nM of MitoTracker Green FM (Life Technologies, CA, USA) for 20 min at 37°C. Cells were washed twice with phenol red-free medium. All live cell imaging experiments were performed in a live cell imaging chamber in the presence of 5% CO_2_ at 37°C using the confocal laser microscope LSM 700 (Zeiss Canada, North York, ON, Canada). The LSM 700 is equipped with the PC running LSM ZEN 2012 (Version 8) software. Mitochondria were visualized using laser 488 power (3-6%) and the plan-Apochromat 40×/1.4 or 63×/1.4 NA oil immersion objectives. At least ten images, z-stacks or time-lapse images were obtained for each cell line. The Fiji software [56] was used to adjust the intensity, split frames and crop images. Mitochondrial length was measured as described previously [57]. For quantitation, measurements were done using the ZEN 2012 software and at least 94 cells were analysed per experimental condition.

### Statistical analysis

Student's *t*-test was performed for statistical analyses. Data are presented as the mean ± standard deviation (SD).
